# Indocyanine green fluorescence-guided laparoscopic colorectal cancer surgery with prophylactic retrograde transileal conduit ureteral catheter placement after previous total cystectomy: a case report

**DOI:** 10.1186/s40792-021-01153-3

**Published:** 2021-03-12

**Authors:** Teppei Kamada, Yuichi Nakaseko, Masashi Yoshida, Wataru Kai, Junji Takahashi, Keigo Nakashima, Norihiko Suzuki, Hironori Ohdaira, Eigoro Yamanouchi, Yutaka Suzuki

**Affiliations:** 1 Department of Surgery, International University of Health and Welfare Hospital, 537-3, Iguchi Nasushiobara, Tochigi 329-2763 Japan; 2 Department of Radiology, International University of Health and Welfare Hospital, Iguchi, Nasushiobara, Tochigi 329-2763 Japan

**Keywords:** Ureteral injury, Ileal conduit, Ureteral stent placement, Indocyanine green

## Abstract

**Background:**

Iatrogenic ureteral injury (UI) is a potentially serious complication of colorectal cancer surgery. Performing perioperative placement of ureteral stents or intraoperative fluorescence navigation surgery for the ureter using indocyanine green (ICG) has been employed as a method of preventing UI. However, transileal conduit stent placement has been considered challenging because it is difficult to identify the ureteral orifice due to the anatomical changes caused by a previous surgery. We report a case in which laparoscopic colectomy was safely performed using a combination of prophylactic transileal conduit ureteral catheter placement and intraoperative ICG fluorescence navigation surgery.

**Case presentation:**

A 75-year-old man presented to our hospital complaining of vomiting and abdominal distension. He had a history of open total cystectomy and ileal conduit urinary diversion 11 years prior to admission. Computed tomography confirmed colon dilation with fecal impaction from the ascending colon to the sigmoid colon and wall thickening in the sigmoid colon. Colonoscopy during the transanal ileus tube insertion revealed a Borrmann type II tumor with circumferential stenosis 10 cm distal to the junction between the descending colon and the sigmoid colon. The patient was diagnosed with colorectal ileus due to obstructive sigmoid colon cancer and underwent transanal ileus tube insertion. Severe intra-abdominal adhesions were expected due to the previous total cystectomy, and the left ureter was near the sigmoid colon tumor; therefore, prophylactic retrograde transileal conduit ureteral catheter placement was performed one day before the elective surgery.

During the operation, 20 ml (5.0 × 10^–2^ mg/ml) ICG was administered from the transileal conduit ureteral catheter, and ICG fluorescence of the ureter was observed in the retroperitoneum. Laparoscopic Hartmann's operation was successfully performed, confirming ureter fluorescence. The operation time was 231 min, with 5 mL of intraoperative bleeding. The ureteral catheter was removed 3 days after the operation. The patient’s postoperative course was good with no complications, and he was discharged on postoperative day 7.

**Conclusions:**

Prophylactic transileal conduit ureteral catheter placement and ICG fluorescence navigation surgery were effective in performing laparoscopic colorectal surgery with severe adhesions after urinary diversion.

## Background

Iatrogenic ureteral injury (UI) is a potentially serious complication of colorectal surgery. The incidence of UI during abdominal surgery is estimated to be between 0.3 and 1.5% [1–4]. Patients with UI usually have complications, including sepsis, renal failure, ureterocutaneous fistula, ureteral strictures, and even the need for reoperation or nephrectomy, which results in perioperative death [5].

Perioperative placement of ureteral stents has been employed as a method of preventing UI. Preoperative prophylactic placement of ureteral stents is performed transurethrally using a cystoscope and is widely known for its usefulness; however, palpation of ureteral stents is difficult in laparoscopic surgery. Recently, the usefulness of intraoperative fluorescence navigation surgery for ureters using indocyanine green (ICG) and methylene blue (MB) has been reported [6–8].

Cases in which ureteral stent placement is difficult include those that are to be performed after urinary diversion. The ileal conduit urinary diversion has been widely used as the most common urinary diversion for more than half a century. However, transileal conduit stent placement has been considered challenging because it is difficult to identify the ureteral orifice due to the anatomical changes caused by a previous surgery. In colorectal surgery after urinary diversion, the ureteral anatomy is often changed due to the adhesions from the previous surgery. Such an operation is extremely high risk for UI; therefore, accurate identification of the ureteral anatomy is essential.

There have been no reports of performing prophylactic placement of ureteral stent after urinary diversion and ICG fluorescence navigation surgery for colorectal surgery.

Herein, we present a case in which a combination of prophylactic placement of a transileal conduit ureteral catheter and intraoperative ICG fluorescence navigation surgery was performed, and laparoscopic colectomy was safely performed.

## Case presentation

**Fig. 1:**
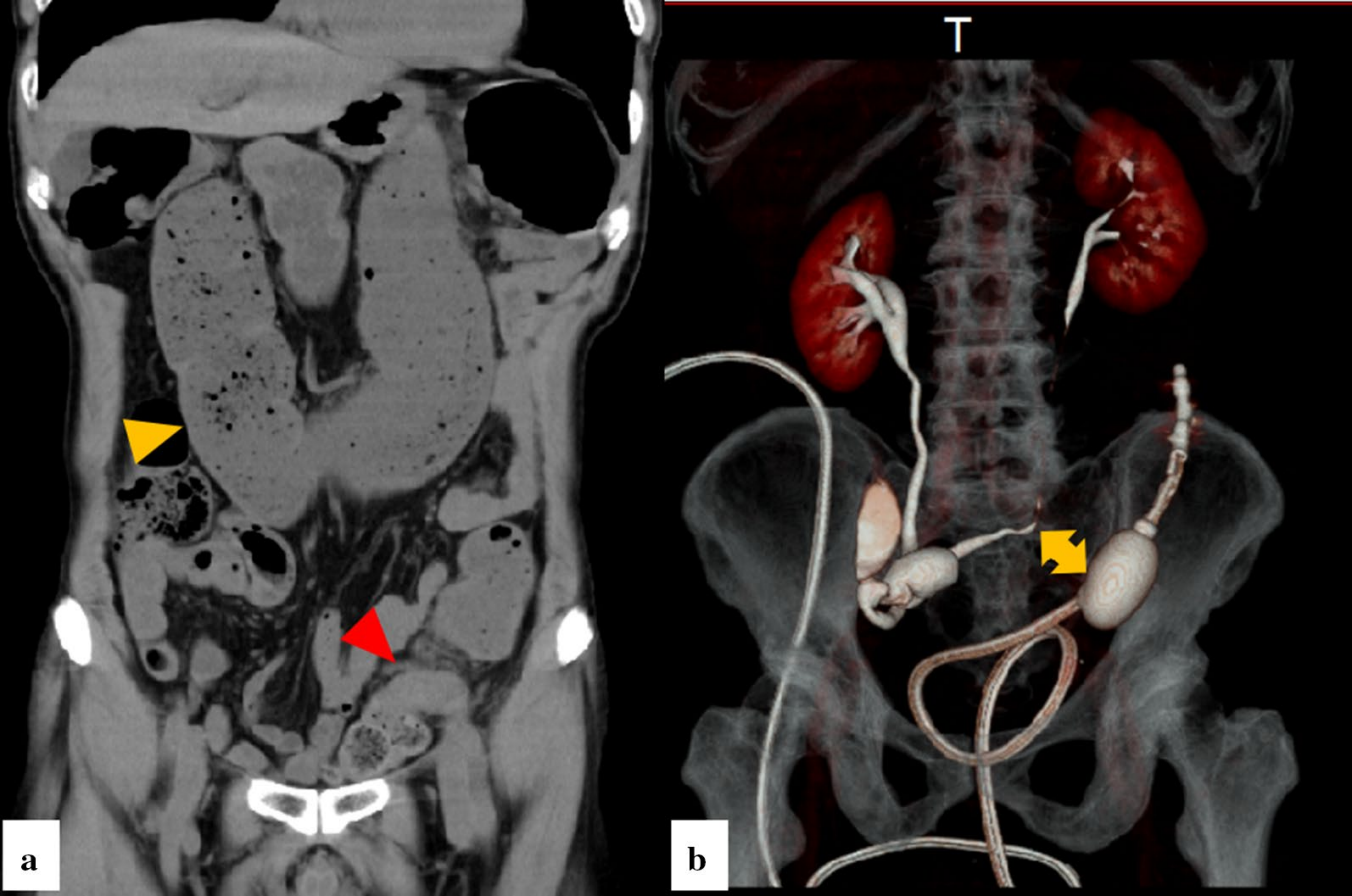
Preoperative computed tomography. **a** Computed tomography (CT) confirms colon dilation with fecal impaction from the ascending colon to the sigmoid colon (yellow arrowhead) and wall thickening in the sigmoid colon (red arrowhead). **b** CT colonography demonstrates that the left ureter was near the sigmoid tumor (yellow arrow)

A 75-year-old man presented to our hospital complaining of vomiting and abdominal distension. Eleven years ago, he was diagnosed with bladder cancer and had undergone open total cystectomy and ileal conduit urinary diversion. Physical examination findings revealed abdominal distension, a midline incision in the lower abdomen, a widespread abdominal incisional hernia that measured 25 cm × 20 cm in the lower left abdomen, and an ileal conduit urinary diversion in the lower right abdomen. On admission, no abnormalities were found on blood examinations. Computed tomography (CT) confirmed colon dilation with fecal impaction from the ascending colon to the sigmoid colon and wall thickening in the sigmoid colon (Fig. 1a). The patient was urgently hospitalized with a diagnosis of colorectal ileus and underwent transanal ileus tube insertion. Colonoscopy during the transanal ileus tube insertion revealed a Borrmann type II tumor with circumferential stenosis 10 cm distal to the junction between the descending colon and the sigmoid colon. With the diagnosis of colonic ileus due to obstructive sigmoid colon cancer, decompression with a transanal ileus tube was performed to manage the obstructive enteritis that persisted for 2 weeks, and an elective surgery was planned. Severe intra-abdominal adhesions were expected due to the previous total cystectomy, and the left ureter was near the sigmoid tumor (Fig. 1b); therefore, prophylactic retrograde transileal conduit ureteral catheter placement before the surgery was planned.

### Procedure of retrograde ureteral catheter placement

The procedure was performed under constant fluoroscopic guidance in the supine position.

**Fig. 2:**
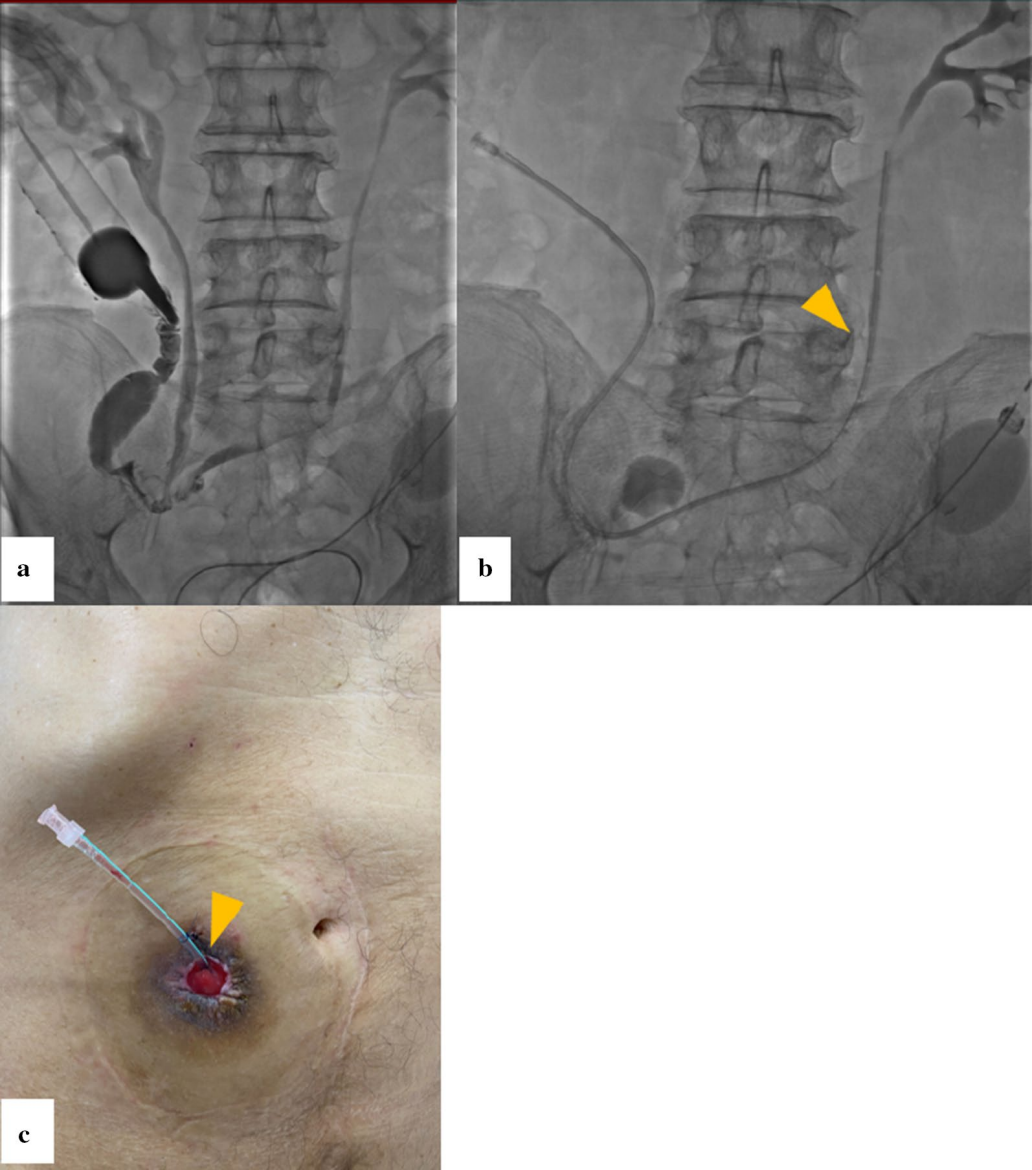
Procedure of retrograde transileal conduit ureteral catheter placement. **a** Contrast medium is injected to provide a road map of the ileal conduit to reflux up the ureters. **b** A 10 Fr catheter with multiple side holes is inserted in the left ureter (arrowhead). **c** A catheter is fixed to the skin near the ileal conduit using 3-0 nylon to prevent deviation due to ureteral peristalsis (arrowhead)

The contrast medium was injected to provide a road map of the ileal conduit to reflux up the ureters, which helped guide the access (Fig. 2a). Confirming the imaging of the left ureter, a 10 Fr catheter with multiple side holes (Create Medic Co., Kanagawa, Japan) was inserted into the left ureter using a 0.035-in., 150-cm-long, stiff-angled hydrophilic guidewire (Radifocus, Terumo, Tokyo, Japan) (Fig. 2b). A catheter was fixed to the skin near the ileal conduit using 3-0 nylon to prevent deviation due to ureteral peristalsis (Fig. 2c).

The elective surgical procedure was scheduled for ICG fluorescence-guided laparoscopic Hartmann's operation considering a wide range of abdominal incisional hernias in the lower abdomen, adhesions from previous surgery, residual obstructive enteritis, and ureteral anatomical change. VISIONSENSE® (Medtronic, Minneapolis, MN, USA) was used to observe ICG fluorescence.

### Intraoperative findings

**Fig. 3:**
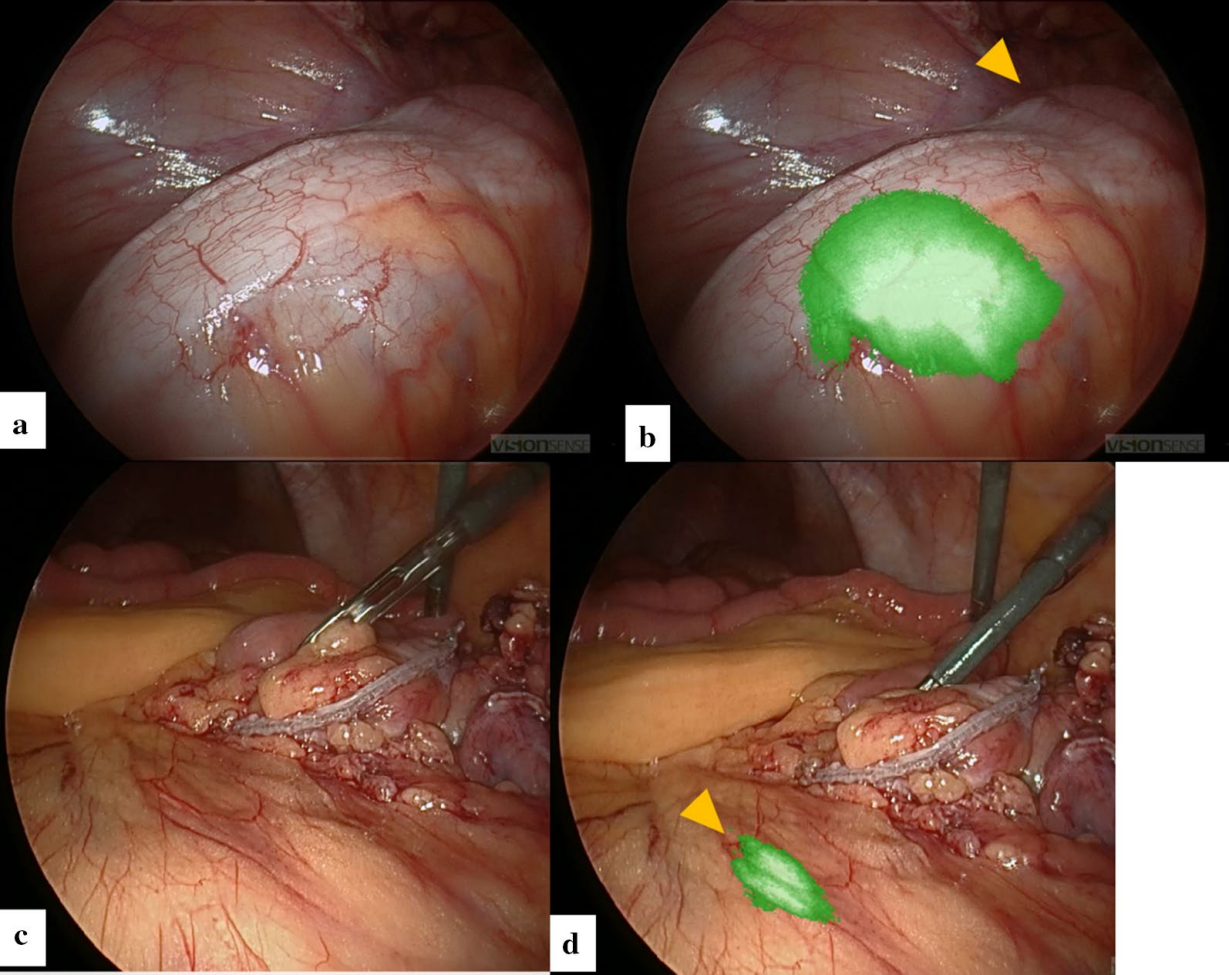
Intraoperative findings. **a** White light image: location of the sigmoid colon cancer is unclear. **b** Full-color near-infrared image. The ICG fluorescence in the transanal ileus tube-tip balloon is confirmed to identify the tumor position. (arrowhead). **c** White light image: the location of the left ureter is unclear. **d** Full-color near-infrared image. ICG fluorescence of the ureter is observed in the retroperitoneum (arrowhead), confirming the preservation of the ureter

Immediately before the operation, the transanal ileus tube was pulled toward the anus, and the balloon was brought close to the obstructive tumor. Intraperitoneal observation revealed extensive small intestinal adhesions on the lower abdominal wall and mesentery of the sigmoid colon. A 4-trocar technique was used for operation (12 mm, 12 mm, 12 mm, and 5 mm trocars). ICG (Diagnogreen®; Dai-Ichi Sankyo Pharm, Tokyo, Japan) at a dose of 20 mL (5.0 μg/mL) was injected into the transanal ileus tube-tip balloon, and the ICG fluorescence in the balloon was confirmed to identify the tumor position (Fig. 3a,b).

The sigmoid colon was mobilized from the retroperitoneum using a lateral approach. Due to severe adhesion of the small intestine, confirming the ICG fluorescence of the ureter was not possible at this time. After mobilization and transection of the sigmoid colon, ICG was administered at a dose of 20 mL (5.0 × 10^–2^ mg/mL) from the transileal conduit ureteral catheter, and the catheter was clamped. ICG fluorescence of the ureter was observed in the retroperitoneum, confirming the preservation of the ureter (Fig. 3c,d). Then, an end-sigmoid colostomy was created in the upper left abdomen, and the operation was completed. The operation time was 231 min, with 5 mL of intraoperative bleeding. The ureteral catheter was removed 3 days after the operation. The patient’s postoperative course was good with no complications, and he was discharged on postoperative day 7. The pathological diagnosis was well-differentiated adenocarcinoma, T3, N0, M0, stage IIa, and postoperative adjuvant chemotherapy was planned.

## Discussion

Iatrogenic UI is a potentially serious complication of abdominal operation, which results in perioperative death or medical litigation [8]. The overall incidence of iatrogenic UI is estimated to be between 0.3 and 1.5%, with 9% of all UIs occurring during colorectal surgery [9]. Unfortunately, many UIs are diagnosed postoperatively [10], and if UIs can be diagnosed accurately during the primary operation, immediate ureteral repair is possible, and outcomes can be expected to improve [11].

Prophylactic ureteral stent placement is a common way to reduce the risk of UI, which plays an important role in the early recognition of the ureteral anatomy and early detection and repair of UI [12].

During laparotomy, palpation and inspection of the ureteral stent can be performed; however, in laparoscopic surgery, only inspection is possible, and conventional ureteral stent placement is not effective. In recent years, the usefulness of ureteral fluorescence navigation surgery using ICG or MB for the prevention of UIs has been reported.

Siddighi et al. stated that intraureteral injection of ICG and visualization under near-infrared (NIR) light allowed for real-time delineation of the ureter in 10 cases during robot-assisted laparoscopic sacrocolpopexy [6]. Lee et al. described that identification of the ureter was successfully performed in 25 cases by using intraureteral injection of ICG and subsequent visualization under near-infrared fluorescence during robot-assisted ureteral reconstructions for various ureteral pathologies [7]. In addition, Barnes et al. reported that ureteral fluorescence was confirmed, and the ureter could be identified in 91.3% of cases by intravenous MB administration in 40 cases of colorectal surgery (laparoscopic, 36 cases; laparotomy, 4 cases) [8].

Usually, preoperative ureteral stent placement is performed transurethrally using a cystoscope; however, cases in which ureteral stent placement is difficult include those that are to be performed after urinary diversion. It is difficult to identify the ureteral orifice owing to its anatomical features, and it is generally considered challenging to place a ureteral stent after ileal conduit urinary diversion.

Charles et al. reported that retrograde transileal conduit stent placement was successfully performed in 18 of 20 (90%) patients with ureteral stenosis, and technical failures were found to be associated with increased length of the ileal conduit (> 20 cm) and ileal conduit kink (< 90 degrees) [13]. Although there have been reports of successful transileal conduit stent placement in the field of urology, there have been no reports of preoperative prophylactic ureteral catheter placement in cases of colorectal cancer after urinary diversion.

In this case, after total laparotomy and ileal conduit urinary diversion, a severe adhesion was expected in the abdominal cavity, and preoperative contrast-enhanced CT showed that the left ureter was near the sigmoid colon tumor. Therefore, we considered that this case was high risk for UI, and preoperative prophylactic ureteral catheter placement was considered desirable.

A 10 Fr catheter was chosen considering the ease of palpation if open conversion occurred because of the excessive adhesion. In addition, we used a catheter with multiple side holes and clamped it after the injection of ICG, which enabled the ureter to be filled with ICG sufficiently during intraoperative ureteral identification. Furthermore, since the ureteral catheter was placed one day before surgery, the catheter was fixed to the skin near the ileal conduit to avoid deviating from ureteral peristalsis.

During the operation, after the sigmoid colon was transected and mobilized, ICG was injected from the ureteral catheter, and ureteral fluorescence was identified in the retroperitoneum; we confirmed that the ureter was preserved. Even if ureteral ICG fluorescence cannot be confirmed, transileal conduit ureteral catheter placement is considered to be highly useful because it is also possible to confirm the preservation of the ureter by administering a contrast medium from the ureteral catheter with intraoperative fluoroscopy.

Recently, some researchers have suggested that a lighted ureteral stent is useful for preventing UI. [14] However, it is more expensive, and there are no reports describing its use after urinary diversion. As mentioned above, our new multipurpose technique is simpler and economical.

Preoperative consultation with a urologist is not required for the above procedures, and there is no effect on the operation time or length of hospital stay, which is a problem when performing a conventional ureteral stent placement [14–16]. Postoperative removal of the catheter was also easily possible at the bedside. We believe that complications, such as urinary tract infection, dysuria, and hematuria associated with ureteral catheter placement [17], can be avoided by removing the catheter early after surgery.

Furthermore, ICG injection into the transanal ileus tube-tip balloon to detect the tumor location is a novel technique. In advanced cancers, the location of the tumor can often be identified macroscopically. However, sometimes the position of tumors, with circumferential stenosis and a depth of T2 or T3, cannot be identified macroscopically. In fact, in this case as well, as shown in Fig. 3a, the ICG injection into the ileus tube-tip balloon is considered to be useful because there was no serosal invasion and, even though it was a T3 tumor, the tumor position could not be identified macroscopically. That is why this technique is performed for T2 or T3 tumors in our institution.

Retrograde transileal conduit ureteral catheter placement might be difficult in cases with increased length of the ileal conduit or ileal conduit kink.

## Conclusions

Prophylactic transileal conduit ureteral catheter placement and ICG fluorescence navigation surgery were effective in performing a laparoscopic colorectal surgery with severe adhesions after urinary diversion.

## Data Availability

Data sharing is not applicable to this article as no datasets were generated or analyzed during the current study.

## Abbreviations

UI: Ureteral injury
ICG: Indocyanine green
MB: Methylene blue
CT: Computed tomography
NIR: Near-infrared

## References

[1] Assimos DG, Patterson LC, Taylor CL (1994). Changing incidence and etiology of iatrogenic ureteral injuries. J Urol.

[2] Larach SW, Patankar SK, Ferrara A, Williamson PR, Perozo SE, Lord AS (1997). Complications of laparoscopic colorectal surgery. Analysis and comparison of early vs. latter experience. Dis Colon Rectum..

[3] Palaniappa NC, Telem DA, Ranasinghe NE, Divino CM (2012). Incidence of iatrogenic ureteral injury after laparoscopic colectomy. Arch Surg.

[4] Halabi WJ, Jafari MD, Nguyen VQ, Carmichael JC, Mills S, Pigazzi A (2014). Ureteral injuries in colorectal surgery: an analysis of trends, outcomes, and risk factors over a 10-year period in the United States. Dis Colon Rectum.

[5] Zafar SN, Ahaghotu CA, Libuit L, Ortega G, Coleman PW, Cornwell EE (2014). Ureteral injury after laparoscopic versus open colectomy. JSLS.

[6] Siddighi S, Yune JJ, Hardesty J (2014). Indocyanine green for intraoperative localization of ureter. Am J Obstet Gynecol.

[7] Lee Z, Moore B, Giusto L, Eun DD (2015). Use of indocyanine green during robot-assisted ureteral reconstructions. Eur Urol.

[8] Barnes TG, Hompes R, Birks J, Mortensen NJ, Jones O, Lindsey I (2018). Methylene blue fluorescence of the ureter during colorectal surgery. Surg Endosc.

[9] Elliott SP, McAninch JW (2006). Ureteral injuries: external and iatrogenic. Urol Clin North Am..

[10] Burks FN, Santucci RA (2014). Management of iatrogenic ureteral injury. Ther Adv Urol.

[11] Sakellariou P, Protopapas AG, Voulgaris Z, Kyritsis N, Rodolakis A, Vlachos G (2002). Management of ureteric injuries during gynecological operations: 10 years experience. Eur J Obstet Gynecol Reprod Biol.

[12] Pokala N, Delaney CP, Kiran RP, Bast J, Angermeier K, Fazio VW (2007). A randomized controlled trial comparing simultaneous intra-operative vs sequential prophylactic ureteric catheter insertion in re-operative and complicated colorectal surgery. Int J Colorectal Dis.

[13] Tapping CR, Briggs JH, Little MW, Bratby MJ, Phillips-Hughes J, Crew JP (2014). Retrograde transileal conduit stent placement for obstructed uropathy–success of primary and exchange stent placement. J Vasc Interv Radiol.

[14] Boyan WP, Lavy D, Dinallo A, Otero J, Roding A, Hanos D (2017). Lighted ureteral stents in laparoscopic colorectal surgery; a five-year experience. Ann Transl Med.

[15] da Silva G, Boutros M, Wexner SD (2012). Role of prophylactic ureteric stents in colorectal surgery. Asian J Endosc Surg.

[16] Speicher PJ, Goldsmith ZG, Nussbaum DP, Turley RS, Peterson AC, Mantyh CR (2014). Ureteral stenting in laparoscopic colorectal surgery. J Surg Res.

[17] Merola J, Arnold B, Luks V, Ibarra C, Resio B, Davis KA (2018). Prophylactic ureteral stent placement vs no ureteral stent placement during open colectomy. JAMA Surg.

